# 4-[3-(Biphenyl-4-yl)-1-phenyl-4,5-di­hydro-1*H*-pyrazol-5-yl]-3-(4-meth­oxy­phen­yl)-1-phenyl-1*H*-pyrazole dioxane monosolvate

**DOI:** 10.1107/S1600536812009117

**Published:** 2012-03-07

**Authors:** Hoong-Kun Fun, Suhana Arshad, Shridhar Malladi, Arun M. Isloor, Kammasandra Nanjunda Shivananda

**Affiliations:** a X-ray Crystallography Unit, School of Physics, Universiti Sains Malaysia, 11800 USM, Penang, Malaysia; b Department of Chemistry, National Institute of Technology-Karnataka, Surathkal, Mangalore 575 025, India; c Schulich faculty of Chemistry, Technion Israel Institute of Technology, Haifa, Israel 32000

## Abstract

In the title compound, C_37_H_30_N_4_O·C_4_H_8_O_2_, the dihedral angle between the pyrazole and dihydro­pyrazole rings is 74.09 (10)°. In the crystal, the components are linked into centrosymmetric tetra­mers (two main mol­ecules and two solvent mol­ecules) by C—H⋯O hydrogen bonds. C—H⋯π and π–π [shortest centroid-centroid separation = 3.6546 (9) Å] inter­actions are also observed.

## Related literature

For the biological and pharmacological activity of pyrazolines, see, for example: Sahu *et al.* (2008 ▶). For ring conformations, see: Cremer & Pople (1975 ▶). For the stability of the temperature controller used in the data collection, see: Cosier & Glazer (1986 ▶). For standard bond lengths, see: Allen *et al.* (1987 ▶).
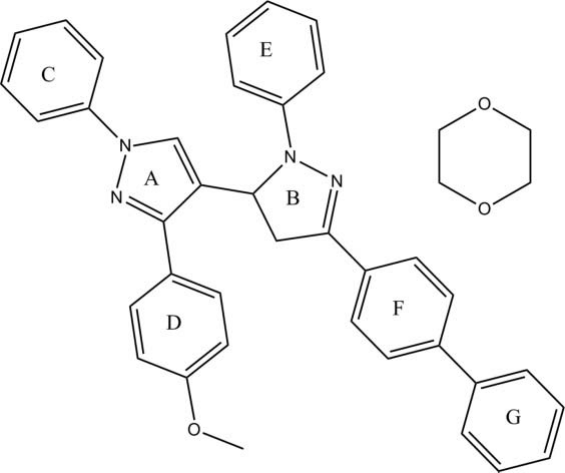


## Experimental

### 

#### Crystal data

C_37_H_30_N_4_O·C_4_H_8_O_2_
*M* *_r_* = 634.75
Triclinic, 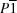

*a* = 11.1189 (2) Å
*b* = 13.0541 (2) Å
*c* = 13.0852 (2) Å
α = 117.309 (1)°
β = 90.468 (1)°
γ = 98.558 (1)°
*V* = 1662.48 (5) Å^3^
*Z* = 2
Mo *K*α radiation
μ = 0.08 mm^−1^
*T* = 100 K
0.26 × 0.19 × 0.05 mm

#### Data collection

Bruker SMART APEXII CCD diffractometer
Absorption correction: multi-scan (*SADABS*; Bruker, 2009 ▶) *T*_min_ = 0.979, *T*_max_ = 0.996
31208 measured reflections
9701 independent reflections
5913 reflections with *I* > 2σ(*I*)
*R*_int_ = 0.046

#### Refinement

*R*[*F* ^2^ > 2σ(*F* ^2^)] = 0.056
*wR*(*F* ^2^) = 0.126
*S* = 1.01
9701 reflections
433 parameters
H-atom parameters constrained
Δρ_max_ = 0.32 e Å^−3^
Δρ_min_ = −0.27 e Å^−3^

### 

Data collection: *APEX2* (Bruker, 2009 ▶); cell refinement: *SAINT* (Bruker, 2009 ▶); data reduction: *SAINT*; program(s) used to solve structure: *SHELXTL* (Sheldrick, 2008 ▶); program(s) used to refine structure: *SHELXTL*; molecular graphics: *SHELXTL*; software used to prepare material for publication: *SHELXTL* and *PLATON* (Spek, 2009 ▶).

## Supplementary Material

Crystal structure: contains datablock(s) global, I. DOI: 10.1107/S1600536812009117/hb6659sup1.cif

Structure factors: contains datablock(s) I. DOI: 10.1107/S1600536812009117/hb6659Isup2.hkl

Supplementary material file. DOI: 10.1107/S1600536812009117/hb6659Isup3.cml

Additional supplementary materials:  crystallographic information; 3D view; checkCIF report

## Figures and Tables

**Table 1:** Hydrogen-bond geometry (Å, °) *Cg*1, *Cg*3 and *Cg*5 are the centroids of the N1/N2/C7/C14/C16, C1–C6 and C32–C37 rings, respectively.

*D*—H⋯*A*	*D*—H	H⋯*A*	*D*⋯*A*	*D*—H⋯*A*
C14—H14*A*⋯O2	0.95	2.28	3.202 (2)	164
C41—H41*B*⋯O1^i^	0.99	2.54	3.344 (3)	139
C1—H1*A*⋯*Cg*1^i^	0.95	2.88	3.412 (2)	117
C33—H33*A*⋯*Cg*3^ii^	0.95	2.79	3.6748 (19)	155
C35—H35*A*⋯*Cg*1^iii^	0.95	2.82	3.684 (2)	151
C41—H41*A*⋯*Cg*5^iv^	0.99	2.83	3.682 (2)	145

Symmetry codes: (i) 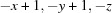; (ii) 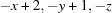; (iii) 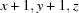; (iv) 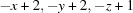.

**Fig. 1.**
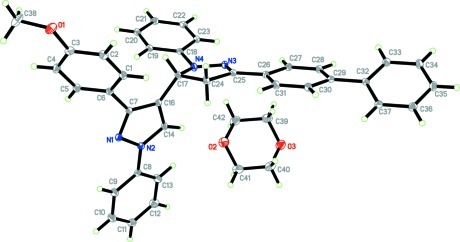
The molecular structure of the title compound, showing 30% probability displacement ellipsoids.

**Fig. 2.**
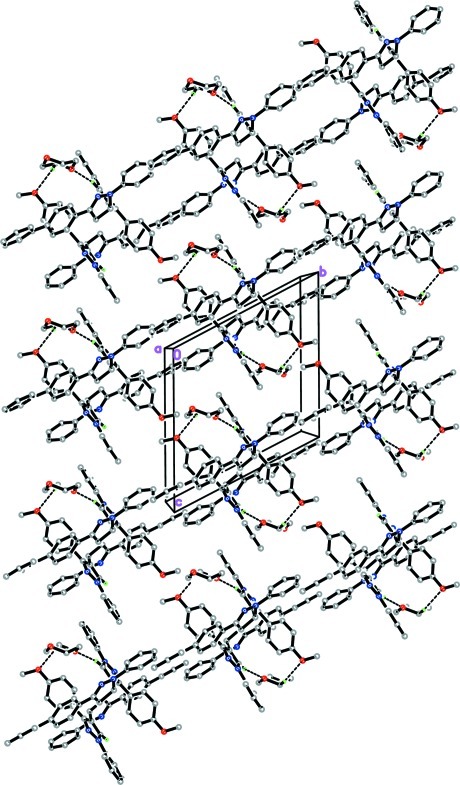
The crystal packing of the title compound. For the sake of clarity, those H atoms not involved in the intermolecular interactions (dashed lines) have been omitted.

## supplementary crystallographic information

### Comment

Pyrazolines are well-known and important nitrogen containing five-membered heterocyclic compounds with biological and pharmacological activities such as analgesic properties (Sahu *et al.*, 2008). As part of our investigations of this area, we have synthesized the title compound to study its crystal structure (Fig. 1).

The solvent 1,4-dioxane ring (O2/O3/C39–C42) adopts a chair conformation (Cremer & Pople, 1975) with puckering parameters Q= 0.567 (2) Å, Θ= 179.0 (2)° and Φ= 302 (6)°. The ring A (N1/N2/C7/C14/C16), B (N3/N4/C17/C24/C25), C (C1–C6), D (C8–C13), E (C18–C23), F (C26–C31) and G (C32–C37) are essentially planar. The dihedral angle between the least-square planes of the rings are A/B = 74.09 (10)°, A/C = 42.50 (10)°, A/D = 8.04 (11)°, A/E = 86.29 (9)°, A/F = 77.25 (9)°, A/G = 83.37 (9)°, B/C = 55.81 (8)°, B/D = 74.18 (10)°, B/E = 19.64 (8)°, B/F = 3.18 (8)°, B/G = 30.67 (8)°, C/D = 49.32 (9)°, C/E = 71.40 (8)°, C/F = 57.94 (8)°, C/G = 86.47 (8)°, D/E = 86.48 (9)°, D/F = 77.36 (9)°, D/G = 86.35 (9)°, E/F = 16.50 (7)°, E/F = 20.45 (7)° and F/G = 28.72 (7)°.

The crystal structure is shown in Fig. 2. The molecules are linked into centrosymmetric tetramers (two main molecules and two solvent molecules) *via* C14—H14A···O2 and C41—H41B···O1 hydrogen bonds (Table 1). C—H···π interactions (Table 1) and π···π interactions of *Cg*2···*Cg*1 = 3.6546 (9) Å (symmetry code: x,y,z) and *Cg*2···*Cg*4 = 3.7773 (10) Å (symmetry code: 2-x,1-y,-z) further stabilized the crystal structure. [*Cg*1, *Cg*2 , *Cg*3, *Cg*4 and *Cg*5 are the centroids of the N1/N2/C7/C14/C16, N3/N4/C17/C24/C25, C1–C6, C26–C31 and C32–C37 rings, respectively].

### Experimental

A mixture of (*E*)-1-(biphenyl-4-yl)-3-(3-(4-methoxyphenyl)-1-phenyl-1 *H*-pyrazol-4-yl)prop-2-en-1-one (0.456 g, 1.0 mmol) and phenylhydrazine (0.162 g, 1.5 mmol) was refluxed in glacial acetic acid for 4 h. The mixture was then cooled to room temperature and resulting solid was filtered and dried to get title compound. Yield: 0.31 g, 56.77%. *M.p.*: 437–439 K. 1,4-Dioxane was used as crystallization solvent to yield colourless plates.

### Refinement

The H atoms were positioned geometrically [C–H = 0.95–0.99 Å] and refined using a riding model with *U*_iso_(H) = 1.2 or 1.5 *U*_eq_(C). A rotating group model was applied to the methyl group.

### Crystal data

**Table:**

C_37_H_30_N_4_O·C_4_H_8_O_2_	*Z* = 2
*M**_r_* = 634.75	*F*(000) = 672
Triclinic, *P*1	*D*_x_ = 1.268 Mg m^−^^3^
Hall symbol: -P 1	Mo *K*α radiation, λ = 0.71073 Å
*a* = 11.1189 (2) Å	Cell parameters from 6011 reflections
*b* = 13.0541 (2) Å	θ = 2.3–29.9°
*c* = 13.0852 (2) Å	µ = 0.08 mm^−^^1^
α = 117.309 (1)°	*T* = 100 K
β = 90.468 (1)°	Plate, colourless
γ = 98.558 (1)°	0.26 × 0.19 × 0.05 mm
*V* = 1662.48 (5) Å^3^	

### Data collection

**Table:**

Bruker SMART APEXII CCD diffractometer	9701 independent reflections
Radiation source: fine-focus sealed tube	5913 reflections with *I* > 2σ(*I*)
Graphite monochromator	*R*_int_ = 0.046
φ and ω scans	θ_max_ = 30.1°, θ_min_ = 1.8°
Absorption correction: multi-scan (*SADABS*; Bruker, 2009)	*h* = −15→15
*T*_min_ = 0.979, *T*_max_ = 0.996	*k* = −18→18
31208 measured reflections	*l* = −17→18

### Refinement

**Table:**

Refinement on *F*^2^	Primary atom site location: structure-invariant direct methods
Least-squares matrix: full	Secondary atom site location: difference Fourier map
*R*[*F*^2^ > 2σ(*F*^2^)] = 0.056	Hydrogen site location: inferred from neighbouring sites
*wR*(*F*^2^) = 0.126	H-atom parameters constrained
*S* = 1.01	*w* = 1/[σ^2^(*F*_o_^2^) + (0.0464*P*)^2^ + 0.3385*P*] where *P* = (*F*_o_^2^ + 2*F*_c_^2^)/3
9701 reflections	(Δ/σ)_max_ < 0.001
433 parameters	Δρ_max_ = 0.32 e Å^−^^3^
0 restraints	Δρ_min_ = −0.27 e Å^−^^3^

### Special details

**Table:**

Experimental. The crystal was placed in the cold stream of an Oxford Cryosystems Cobra open-flow nitrogen cryostat (Cosier & Glazer, 1986) operating at 100.0 (1) K.
Geometry. All e.s.d.'s (except the e.s.d. in the dihedral angle between two l.s. planes) are estimated using the full covariance matrix. The cell e.s.d.'s are taken into account individually in the estimation of e.s.d.'s in distances, angles and torsion angles; correlations between e.s.d.'s in cell parameters are only used when they are defined by crystal symmetry. An approximate (isotropic) treatment of cell e.s.d.'s is used for estimating e.s.d.'s involving l.s. planes.
Refinement. Refinement of *F*^2^ against ALL reflections. The weighted *R*-factor *wR* and goodness of fit *S* are based on *F*^2^, conventional *R*-factors *R* are based on *F*, with *F* set to zero for negative *F*^2^. The threshold expression of *F*^2^ > σ(*F*^2^) is used only for calculating *R*-factors(gt) *etc*. and is not relevant to the choice of reflections for refinement. *R*-factors based on *F*^2^ are statistically about twice as large as those based on *F*, and *R*- factors based on ALL data will be even larger.

### Fractional atomic coordinates and isotropic or equivalent isotropic displacement parameters (Å^2^)

**Table:**

	*x*	*y*	*z*	*U*_iso_*/*U*_eq_	
O1	0.38654 (11)	0.04602 (10)	−0.41845 (10)	0.0311 (3)	
N1	0.39934 (11)	0.40181 (11)	0.12580 (11)	0.0175 (3)	
N2	0.46058 (11)	0.47467 (11)	0.23135 (10)	0.0168 (3)	
N3	0.92168 (11)	0.51162 (11)	0.16922 (11)	0.0191 (3)	
N4	0.82182 (11)	0.42528 (11)	0.14802 (11)	0.0195 (3)	
C1	0.51535 (14)	0.30555 (14)	−0.15631 (13)	0.0195 (3)	
H1A	0.5745	0.3739	−0.1354	0.023*	
C2	0.48901 (14)	0.22438 (14)	−0.27172 (14)	0.0220 (3)	
H2A	0.5292	0.2379	−0.3293	0.026*	
C3	0.40379 (14)	0.12316 (14)	−0.30321 (13)	0.0216 (3)	
C4	0.34192 (14)	0.10595 (14)	−0.21927 (14)	0.0227 (4)	
H4A	0.2815	0.0384	−0.2405	0.027*	
C5	0.36886 (14)	0.18822 (13)	−0.10394 (13)	0.0200 (3)	
H5A	0.3265	0.1758	−0.0467	0.024*	
C6	0.45637 (13)	0.28837 (13)	−0.07028 (13)	0.0165 (3)	
C7	0.48691 (13)	0.37256 (13)	0.05328 (13)	0.0163 (3)	
C8	0.39562 (14)	0.52468 (13)	0.33118 (13)	0.0188 (3)	
C9	0.26970 (15)	0.51084 (15)	0.32004 (15)	0.0306 (4)	
H9A	0.2259	0.4682	0.2460	0.037*	
C10	0.20800 (16)	0.56008 (17)	0.41854 (16)	0.0397 (5)	
H10A	0.1215	0.5506	0.4111	0.048*	
C11	0.26965 (16)	0.62242 (15)	0.52684 (15)	0.0323 (4)	
H11A	0.2264	0.6554	0.5935	0.039*	
C12	0.39533 (16)	0.63607 (16)	0.53661 (15)	0.0315 (4)	
H12A	0.4389	0.6792	0.6107	0.038*	
C13	0.45882 (15)	0.58756 (15)	0.43952 (14)	0.0263 (4)	
H13A	0.5454	0.5974	0.4472	0.032*	
C14	0.58366 (13)	0.49157 (13)	0.22507 (13)	0.0182 (3)	
H14A	0.6438	0.5391	0.2876	0.022*	
C16	0.60449 (13)	0.42740 (13)	0.11198 (13)	0.0168 (3)	
C17	0.72666 (13)	0.41884 (14)	0.06391 (13)	0.0180 (3)	
H17A	0.7214	0.3439	−0.0093	0.022*	
C18	0.83724 (13)	0.33063 (13)	0.16535 (13)	0.0176 (3)	
C19	0.74746 (14)	0.23032 (14)	0.12384 (13)	0.0196 (3)	
H19A	0.6732	0.2274	0.0855	0.024*	
C20	0.76629 (14)	0.13530 (14)	0.13839 (14)	0.0223 (4)	
H20A	0.7057	0.0667	0.1077	0.027*	
C21	0.87245 (15)	0.13869 (15)	0.19716 (14)	0.0240 (4)	
H21A	0.8844	0.0735	0.2077	0.029*	
C22	0.96060 (14)	0.23863 (14)	0.24010 (13)	0.0219 (4)	
H22A	1.0333	0.2419	0.2809	0.026*	
C23	0.94465 (14)	0.33354 (14)	0.22467 (13)	0.0191 (3)	
H23A	1.0065	0.4011	0.2542	0.023*	
C24	0.77997 (13)	0.52319 (14)	0.04252 (14)	0.0219 (3)	
H24A	0.7918	0.4971	−0.0401	0.026*	
H24B	0.7266	0.5832	0.0685	0.026*	
C25	0.90040 (13)	0.56884 (13)	0.11509 (13)	0.0183 (3)	
C26	0.98614 (14)	0.67053 (13)	0.12592 (13)	0.0186 (3)	
C27	1.09754 (14)	0.70889 (14)	0.19448 (13)	0.0197 (3)	
H27A	1.1187	0.6675	0.2338	0.024*	
C28	1.17650 (14)	0.80617 (13)	0.20528 (13)	0.0196 (3)	
H28A	1.2511	0.8308	0.2524	0.023*	
C29	1.14944 (13)	0.86939 (13)	0.14855 (13)	0.0179 (3)	
C30	1.03849 (14)	0.83051 (14)	0.07968 (13)	0.0209 (3)	
H30A	1.0178	0.8715	0.0398	0.025*	
C31	0.95847 (14)	0.73316 (14)	0.06885 (14)	0.0209 (3)	
H31A	0.8837	0.7087	0.0219	0.025*	
C32	1.23501 (14)	0.97374 (13)	0.16014 (13)	0.0181 (3)	
C33	1.36075 (14)	0.98388 (14)	0.18125 (14)	0.0220 (3)	
H33A	1.3919	0.9235	0.1888	0.026*	
C34	1.44036 (15)	1.08082 (14)	0.19126 (14)	0.0251 (4)	
H34A	1.5256	1.0864	0.2057	0.030*	
C35	1.39703 (15)	1.16958 (14)	0.18037 (14)	0.0234 (4)	
H35A	1.4521	1.2358	0.1870	0.028*	
C36	1.27214 (15)	1.16119 (14)	0.15967 (13)	0.0224 (4)	
H36A	1.2415	1.2218	0.1522	0.027*	
C37	1.19241 (14)	1.06422 (13)	0.14991 (13)	0.0201 (3)	
H37A	1.1072	1.0592	0.1360	0.024*	
C38	0.3130 (2)	−0.06576 (16)	−0.45170 (16)	0.0404 (5)	
H38A	0.3075	−0.1126	−0.5359	0.061*	
H38B	0.2310	−0.0552	−0.4264	0.061*	
H38C	0.3501	−0.1061	−0.4156	0.061*	
O2	0.76445 (11)	0.69333 (11)	0.43578 (10)	0.0364 (3)	
O3	0.96457 (11)	0.86884 (11)	0.57046 (11)	0.0367 (3)	
C39	0.98147 (17)	0.75738 (17)	0.48241 (17)	0.0401 (5)	
H39A	1.0561	0.7364	0.5041	0.048*	
H39B	0.9930	0.7615	0.4094	0.048*	
C40	0.85522 (17)	0.89691 (16)	0.53937 (16)	0.0361 (5)	
H40A	0.8634	0.9021	0.4665	0.043*	
H40B	0.8421	0.9740	0.6004	0.043*	
C41	0.74771 (17)	0.80558 (16)	0.52416 (16)	0.0351 (5)	
H41A	0.7377	0.8024	0.5978	0.042*	
H41B	0.6727	0.8265	0.5031	0.042*	
C42	0.87401 (17)	0.66411 (16)	0.46398 (16)	0.0378 (5)	
H42A	0.8874	0.5884	0.4007	0.045*	
H42B	0.8663	0.6554	0.5350	0.045*	

### Atomic displacement parameters (Å^2^)

**Table:**

	*U*^11^	*U*^22^	*U*^33^	*U*^12^	*U*^13^	*U*^23^
O1	0.0427 (7)	0.0263 (7)	0.0191 (6)	0.0054 (6)	0.0009 (5)	0.0065 (5)
N1	0.0167 (6)	0.0149 (7)	0.0202 (6)	0.0016 (5)	−0.0008 (5)	0.0080 (5)
N2	0.0162 (6)	0.0163 (7)	0.0170 (6)	0.0032 (5)	0.0013 (5)	0.0069 (5)
N3	0.0153 (6)	0.0160 (7)	0.0246 (7)	0.0003 (5)	0.0011 (5)	0.0088 (6)
N4	0.0140 (6)	0.0191 (7)	0.0273 (7)	0.0002 (5)	−0.0017 (5)	0.0132 (6)
C1	0.0163 (7)	0.0198 (9)	0.0259 (8)	0.0034 (6)	0.0013 (6)	0.0136 (7)
C2	0.0231 (8)	0.0262 (9)	0.0220 (8)	0.0059 (7)	0.0047 (6)	0.0151 (7)
C3	0.0256 (8)	0.0216 (9)	0.0179 (8)	0.0079 (7)	0.0003 (6)	0.0083 (7)
C4	0.0226 (8)	0.0172 (9)	0.0263 (9)	−0.0004 (7)	−0.0014 (7)	0.0096 (7)
C5	0.0189 (8)	0.0199 (9)	0.0218 (8)	0.0022 (7)	0.0028 (6)	0.0106 (7)
C6	0.0145 (7)	0.0175 (8)	0.0203 (8)	0.0050 (6)	0.0014 (6)	0.0106 (6)
C7	0.0156 (7)	0.0137 (8)	0.0217 (8)	0.0028 (6)	0.0014 (6)	0.0100 (6)
C8	0.0205 (8)	0.0140 (8)	0.0228 (8)	0.0036 (6)	0.0047 (6)	0.0089 (6)
C9	0.0204 (8)	0.0290 (10)	0.0265 (9)	0.0021 (7)	0.0021 (7)	0.0001 (8)
C10	0.0207 (9)	0.0380 (12)	0.0366 (11)	0.0014 (8)	0.0081 (8)	−0.0019 (9)
C11	0.0322 (10)	0.0253 (10)	0.0295 (9)	0.0047 (8)	0.0120 (8)	0.0042 (8)
C12	0.0335 (10)	0.0344 (11)	0.0215 (9)	0.0075 (8)	0.0031 (7)	0.0083 (8)
C13	0.0215 (8)	0.0321 (10)	0.0238 (8)	0.0057 (7)	0.0029 (7)	0.0113 (7)
C14	0.0142 (7)	0.0174 (8)	0.0222 (8)	0.0014 (6)	−0.0015 (6)	0.0091 (6)
C16	0.0158 (7)	0.0152 (8)	0.0209 (8)	0.0025 (6)	0.0003 (6)	0.0097 (6)
C17	0.0143 (7)	0.0190 (8)	0.0210 (8)	0.0022 (6)	0.0005 (6)	0.0098 (6)
C18	0.0163 (7)	0.0182 (8)	0.0195 (7)	0.0053 (6)	0.0063 (6)	0.0091 (6)
C19	0.0152 (7)	0.0210 (9)	0.0229 (8)	0.0043 (6)	0.0035 (6)	0.0101 (7)
C20	0.0187 (8)	0.0202 (9)	0.0296 (9)	0.0027 (7)	0.0074 (7)	0.0131 (7)
C21	0.0249 (8)	0.0235 (9)	0.0314 (9)	0.0097 (7)	0.0093 (7)	0.0174 (8)
C22	0.0189 (8)	0.0260 (9)	0.0232 (8)	0.0079 (7)	0.0040 (6)	0.0123 (7)
C23	0.0160 (7)	0.0194 (8)	0.0209 (8)	0.0022 (6)	0.0017 (6)	0.0087 (7)
C24	0.0161 (7)	0.0230 (9)	0.0302 (9)	0.0010 (7)	−0.0001 (6)	0.0160 (7)
C25	0.0155 (7)	0.0179 (8)	0.0215 (8)	0.0040 (6)	0.0038 (6)	0.0087 (7)
C26	0.0172 (7)	0.0166 (8)	0.0217 (8)	0.0038 (6)	0.0047 (6)	0.0084 (6)
C27	0.0202 (8)	0.0189 (8)	0.0220 (8)	0.0050 (7)	0.0031 (6)	0.0108 (7)
C28	0.0171 (7)	0.0199 (9)	0.0207 (8)	0.0032 (6)	0.0017 (6)	0.0086 (7)
C29	0.0177 (7)	0.0157 (8)	0.0193 (8)	0.0041 (6)	0.0054 (6)	0.0069 (6)
C30	0.0216 (8)	0.0207 (9)	0.0239 (8)	0.0053 (7)	0.0038 (6)	0.0128 (7)
C31	0.0171 (8)	0.0223 (9)	0.0239 (8)	0.0029 (7)	0.0016 (6)	0.0115 (7)
C32	0.0186 (8)	0.0180 (8)	0.0182 (7)	0.0044 (6)	0.0052 (6)	0.0083 (6)
C33	0.0210 (8)	0.0206 (9)	0.0281 (9)	0.0043 (7)	0.0037 (7)	0.0141 (7)
C34	0.0193 (8)	0.0260 (10)	0.0321 (9)	0.0010 (7)	0.0021 (7)	0.0161 (8)
C35	0.0248 (8)	0.0200 (9)	0.0257 (8)	−0.0012 (7)	0.0039 (7)	0.0122 (7)
C36	0.0271 (9)	0.0198 (9)	0.0235 (8)	0.0059 (7)	0.0046 (7)	0.0121 (7)
C37	0.0203 (8)	0.0193 (9)	0.0211 (8)	0.0052 (7)	0.0045 (6)	0.0092 (7)
C38	0.0608 (13)	0.0224 (10)	0.0252 (9)	0.0019 (9)	−0.0082 (9)	0.0020 (8)
O2	0.0327 (7)	0.0361 (8)	0.0291 (7)	0.0064 (6)	−0.0089 (5)	0.0057 (6)
O3	0.0364 (7)	0.0297 (7)	0.0362 (7)	0.0043 (6)	−0.0040 (6)	0.0094 (6)
C39	0.0332 (10)	0.0365 (12)	0.0410 (11)	0.0121 (9)	0.0018 (9)	0.0083 (9)
C40	0.0468 (12)	0.0303 (11)	0.0331 (10)	0.0137 (9)	0.0051 (9)	0.0144 (8)
C41	0.0347 (10)	0.0378 (12)	0.0301 (10)	0.0136 (9)	0.0029 (8)	0.0113 (9)
C42	0.0385 (11)	0.0302 (11)	0.0334 (10)	0.0098 (9)	−0.0065 (8)	0.0044 (8)

### Geometric parameters (Å, º)

**Table:**

O1—C3	1.3672 (18)	C22—C23	1.379 (2)
O1—C38	1.430 (2)	C22—H22A	0.9500
N1—C7	1.3371 (19)	C23—H23A	0.9500
N1—N2	1.3647 (16)	C24—C25	1.508 (2)
N2—C14	1.3627 (18)	C24—H24A	0.9900
N2—C8	1.4238 (19)	C24—H24B	0.9900
N3—C25	1.2848 (19)	C25—C26	1.463 (2)
N3—N4	1.3842 (17)	C26—C31	1.397 (2)
N4—C18	1.3882 (19)	C26—C27	1.405 (2)
N4—C17	1.4857 (18)	C27—C28	1.380 (2)
C1—C2	1.386 (2)	C27—H27A	0.9500
C1—C6	1.396 (2)	C28—C29	1.398 (2)
C1—H1A	0.9500	C28—H28A	0.9500
C2—C3	1.391 (2)	C29—C30	1.403 (2)
C2—H2A	0.9500	C29—C32	1.485 (2)
C3—C4	1.387 (2)	C30—C31	1.387 (2)
C4—C5	1.389 (2)	C30—H30A	0.9500
C4—H4A	0.9500	C31—H31A	0.9500
C5—C6	1.391 (2)	C32—C37	1.396 (2)
C5—H5A	0.9500	C32—C33	1.398 (2)
C6—C7	1.479 (2)	C33—C34	1.384 (2)
C7—C16	1.4185 (19)	C33—H33A	0.9500
C8—C9	1.383 (2)	C34—C35	1.383 (2)
C8—C13	1.386 (2)	C34—H34A	0.9500
C9—C10	1.389 (2)	C35—C36	1.391 (2)
C9—H9A	0.9500	C35—H35A	0.9500
C10—C11	1.379 (2)	C36—C37	1.386 (2)
C10—H10A	0.9500	C36—H36A	0.9500
C11—C12	1.379 (2)	C37—H37A	0.9500
C11—H11A	0.9500	C38—H38A	0.9800
C12—C13	1.387 (2)	C38—H38B	0.9800
C12—H12A	0.9500	C38—H38C	0.9800
C13—H13A	0.9500	O2—C42	1.423 (2)
C14—C16	1.368 (2)	O2—C41	1.431 (2)
C14—H14A	0.9500	O3—C39	1.424 (2)
C16—C17	1.499 (2)	O3—C40	1.428 (2)
C17—C24	1.548 (2)	C39—C42	1.504 (3)
C17—H17A	1.0000	C39—H39A	0.9900
C18—C19	1.398 (2)	C39—H39B	0.9900
C18—C23	1.405 (2)	C40—C41	1.498 (3)
C19—C20	1.383 (2)	C40—H40A	0.9900
C19—H19A	0.9500	C40—H40B	0.9900
C20—C21	1.387 (2)	C41—H41A	0.9900
C20—H20A	0.9500	C41—H41B	0.9900
C21—C22	1.384 (2)	C42—H42A	0.9900
C21—H21A	0.9500	C42—H42B	0.9900
			
C3—O1—C38	117.32 (14)	C25—C24—C17	102.30 (12)
C7—N1—N2	104.59 (11)	C25—C24—H24A	111.3
C14—N2—N1	111.88 (12)	C17—C24—H24A	111.3
C14—N2—C8	127.59 (12)	C25—C24—H24B	111.3
N1—N2—C8	120.51 (12)	C17—C24—H24B	111.3
C25—N3—N4	108.93 (12)	H24A—C24—H24B	109.2
N3—N4—C18	118.82 (12)	N3—C25—C26	121.97 (13)
N3—N4—C17	112.63 (12)	N3—C25—C24	114.21 (14)
C18—N4—C17	123.26 (12)	C26—C25—C24	123.81 (14)
C2—C1—C6	121.03 (15)	C31—C26—C27	118.09 (15)
C2—C1—H1A	119.5	C31—C26—C25	120.55 (14)
C6—C1—H1A	119.5	C27—C26—C25	121.35 (14)
C1—C2—C3	120.01 (15)	C28—C27—C26	120.61 (15)
C1—C2—H2A	120.0	C28—C27—H27A	119.7
C3—C2—H2A	120.0	C26—C27—H27A	119.7
O1—C3—C4	124.08 (15)	C27—C28—C29	121.66 (14)
O1—C3—C2	116.11 (15)	C27—C28—H28A	119.2
C4—C3—C2	119.81 (14)	C29—C28—H28A	119.2
C3—C4—C5	119.56 (15)	C28—C29—C30	117.60 (15)
C3—C4—H4A	120.2	C28—C29—C32	121.40 (13)
C5—C4—H4A	120.2	C30—C29—C32	121.00 (14)
C4—C5—C6	121.54 (15)	C31—C30—C29	121.07 (15)
C4—C5—H5A	119.2	C31—C30—H30A	119.5
C6—C5—H5A	119.2	C29—C30—H30A	119.5
C5—C6—C1	118.01 (14)	C30—C31—C26	120.98 (14)
C5—C6—C7	120.63 (14)	C30—C31—H31A	119.5
C1—C6—C7	121.35 (14)	C26—C31—H31A	119.5
N1—C7—C16	111.38 (13)	C37—C32—C33	118.08 (14)
N1—C7—C6	120.78 (13)	C37—C32—C29	121.07 (13)
C16—C7—C6	127.78 (13)	C33—C32—C29	120.85 (14)
C9—C8—C13	120.04 (15)	C34—C33—C32	120.74 (15)
C9—C8—N2	120.04 (14)	C34—C33—H33A	119.6
C13—C8—N2	119.92 (14)	C32—C33—H33A	119.6
C8—C9—C10	119.15 (16)	C35—C34—C33	120.60 (15)
C8—C9—H9A	120.4	C35—C34—H34A	119.7
C10—C9—H9A	120.4	C33—C34—H34A	119.7
C11—C10—C9	121.42 (17)	C34—C35—C36	119.48 (15)
C11—C10—H10A	119.3	C34—C35—H35A	120.3
C9—C10—H10A	119.3	C36—C35—H35A	120.3
C10—C11—C12	118.80 (17)	C37—C36—C35	119.91 (15)
C10—C11—H11A	120.6	C37—C36—H36A	120.0
C12—C11—H11A	120.6	C35—C36—H36A	120.0
C11—C12—C13	120.78 (16)	C36—C37—C32	121.18 (14)
C11—C12—H12A	119.6	C36—C37—H37A	119.4
C13—C12—H12A	119.6	C32—C37—H37A	119.4
C8—C13—C12	119.80 (16)	O1—C38—H38A	109.5
C8—C13—H13A	120.1	O1—C38—H38B	109.5
C12—C13—H13A	120.1	H38A—C38—H38B	109.5
N2—C14—C16	107.22 (13)	O1—C38—H38C	109.5
N2—C14—H14A	126.4	H38A—C38—H38C	109.5
C16—C14—H14A	126.4	H38B—C38—H38C	109.5
C14—C16—C7	104.93 (13)	C42—O2—C41	109.84 (13)
C14—C16—C17	126.23 (13)	C39—O3—C40	108.80 (13)
C7—C16—C17	128.84 (14)	O3—C39—C42	111.51 (16)
N4—C17—C16	111.32 (12)	O3—C39—H39A	109.3
N4—C17—C24	101.74 (11)	C42—C39—H39A	109.3
C16—C17—C24	114.11 (13)	O3—C39—H39B	109.3
N4—C17—H17A	109.8	C42—C39—H39B	109.3
C16—C17—H17A	109.8	H39A—C39—H39B	108.0
C24—C17—H17A	109.8	O3—C40—C41	110.58 (15)
N4—C18—C19	121.16 (13)	O3—C40—H40A	109.5
N4—C18—C23	120.34 (14)	C41—C40—H40A	109.5
C19—C18—C23	118.50 (14)	O3—C40—H40B	109.5
C20—C19—C18	120.32 (14)	C41—C40—H40B	109.5
C20—C19—H19A	119.8	H40A—C40—H40B	108.1
C18—C19—H19A	119.8	O2—C41—C40	110.46 (15)
C19—C20—C21	120.92 (15)	O2—C41—H41A	109.6
C19—C20—H20A	119.5	C40—C41—H41A	109.6
C21—C20—H20A	119.5	O2—C41—H41B	109.6
C22—C21—C20	118.89 (15)	C40—C41—H41B	109.6
C22—C21—H21A	120.6	H41A—C41—H41B	108.1
C20—C21—H21A	120.6	O2—C42—C39	110.73 (16)
C23—C22—C21	121.12 (14)	O2—C42—H42A	109.5
C23—C22—H22A	119.4	C39—C42—H42A	109.5
C21—C22—H22A	119.4	O2—C42—H42B	109.5
C22—C23—C18	120.22 (15)	C39—C42—H42B	109.5
C22—C23—H23A	119.9	H42A—C42—H42B	108.1
C18—C23—H23A	119.9		
			
C7—N1—N2—C14	0.52 (16)	N3—N4—C18—C19	−166.93 (14)
C7—N1—N2—C8	179.51 (13)	C17—N4—C18—C19	−14.5 (2)
C25—N3—N4—C18	159.61 (13)	N3—N4—C18—C23	12.6 (2)
C25—N3—N4—C17	4.42 (17)	C17—N4—C18—C23	164.99 (14)
C6—C1—C2—C3	−0.8 (2)	N4—C18—C19—C20	177.71 (14)
C38—O1—C3—C4	−9.0 (2)	C23—C18—C19—C20	−1.8 (2)
C38—O1—C3—C2	171.30 (15)	C18—C19—C20—C21	1.9 (2)
C1—C2—C3—O1	−177.80 (13)	C19—C20—C21—C22	−0.8 (2)
C1—C2—C3—C4	2.5 (2)	C20—C21—C22—C23	−0.4 (2)
O1—C3—C4—C5	178.08 (14)	C21—C22—C23—C18	0.5 (2)
C2—C3—C4—C5	−2.2 (2)	N4—C18—C23—C22	−178.93 (14)
C3—C4—C5—C6	0.3 (2)	C19—C18—C23—C22	0.6 (2)
C4—C5—C6—C1	1.3 (2)	N4—C17—C24—C25	2.31 (15)
C4—C5—C6—C7	−177.67 (14)	C16—C17—C24—C25	122.30 (13)
C2—C1—C6—C5	−1.0 (2)	N4—N3—C25—C26	176.31 (13)
C2—C1—C6—C7	177.92 (13)	N4—N3—C25—C24	−2.69 (18)
N2—N1—C7—C16	−0.51 (16)	C17—C24—C25—N3	0.08 (18)
N2—N1—C7—C6	176.81 (13)	C17—C24—C25—C26	−178.90 (14)
C5—C6—C7—N1	−41.4 (2)	N3—C25—C26—C31	−177.90 (15)
C1—C6—C7—N1	139.67 (15)	C24—C25—C26—C31	1.0 (2)
C5—C6—C7—C16	135.43 (16)	N3—C25—C26—C27	1.4 (2)
C1—C6—C7—C16	−43.5 (2)	C24—C25—C26—C27	−179.69 (15)
C14—N2—C8—C9	171.24 (16)	C31—C26—C27—C28	0.4 (2)
N1—N2—C8—C9	−7.6 (2)	C25—C26—C27—C28	−178.92 (14)
C14—N2—C8—C13	−8.6 (2)	C26—C27—C28—C29	−0.4 (2)
N1—N2—C8—C13	172.64 (14)	C27—C28—C29—C30	0.0 (2)
C13—C8—C9—C10	−0.4 (3)	C27—C28—C29—C32	−179.76 (14)
N2—C8—C9—C10	179.84 (17)	C28—C29—C30—C31	0.3 (2)
C8—C9—C10—C11	0.1 (3)	C32—C29—C30—C31	−179.95 (14)
C9—C10—C11—C12	0.3 (3)	C29—C30—C31—C26	−0.2 (2)
C10—C11—C12—C13	−0.4 (3)	C27—C26—C31—C30	−0.1 (2)
C9—C8—C13—C12	0.3 (3)	C25—C26—C31—C30	179.22 (15)
N2—C8—C13—C12	−179.93 (15)	C28—C29—C32—C37	−151.41 (15)
C11—C12—C13—C8	0.1 (3)	C30—C29—C32—C37	28.8 (2)
N1—N2—C14—C16	−0.34 (17)	C28—C29—C32—C33	28.8 (2)
C8—N2—C14—C16	−179.23 (14)	C30—C29—C32—C33	−150.93 (15)
N2—C14—C16—C7	0.01 (16)	C37—C32—C33—C34	−0.2 (2)
N2—C14—C16—C17	−179.81 (14)	C29—C32—C33—C34	179.57 (15)
N1—C7—C16—C14	0.32 (17)	C32—C33—C34—C35	−0.1 (3)
C6—C7—C16—C14	−176.76 (15)	C33—C34—C35—C36	0.3 (2)
N1—C7—C16—C17	−179.86 (14)	C34—C35—C36—C37	−0.1 (2)
C6—C7—C16—C17	3.1 (3)	C35—C36—C37—C32	−0.2 (2)
N3—N4—C17—C16	−126.06 (13)	C33—C32—C37—C36	0.4 (2)
C18—N4—C17—C16	80.03 (18)	C29—C32—C37—C36	−179.42 (14)
N3—N4—C17—C24	−4.11 (16)	C40—O3—C39—C42	−57.7 (2)
C18—N4—C17—C24	−158.03 (14)	C39—O3—C40—C41	58.7 (2)
C14—C16—C17—N4	33.7 (2)	C42—O2—C41—C40	57.6 (2)
C7—C16—C17—N4	−146.12 (15)	O3—C40—C41—O2	−59.5 (2)
C14—C16—C17—C24	−80.80 (19)	C41—O2—C42—C39	−56.2 (2)
C7—C16—C17—C24	99.41 (18)	O3—C39—C42—O2	57.3 (2)

### Hydrogen-bond geometry (Å, º)

*Cg*1, *Cg*3 and *Cg*5 are the centroids of the N1/N2/C7/C14/C16, C1–C6 and C32–C37 rings, respectively.

**Table:**

*D*—H···*A*	*D*—H	H···*A*	*D*···*A*	*D*—H···*A*
C14—H14*A*···O2	0.95	2.28	3.202 (2)	164
C41—H41*B*···O1^i^	0.99	2.54	3.344 (3)	139
C1—H1*A*···*Cg*1^i^	0.95	2.88	3.412 (2)	117
C33—H33*A*···*Cg*3^ii^	0.95	2.79	3.6748 (19)	155
C35—H35*A*···*Cg*1^iii^	0.95	2.82	3.684 (2)	151
C41—H41*A*···*Cg*5^iv^	0.99	2.83	3.682 (2)	145

Symmetry codes: (i) −*x*+1, −*y*+1, −*z*; (ii) −*x*+2, −*y*+1, −*z*; (iii) *x*+1, *y*+1, *z*; (iv) −*x*+2, −*y*+2, −*z*+1.
